# 人肺腺癌TLE1 N端Q结构域片段在原核系统表达纯化及其多克隆抗体的制备

**DOI:** 10.3779/j.issn.1009-3419.2010.11.02

**Published:** 2010-11-20

**Authors:** 苏 王, 志飞 徐, 华 唐, 凌云 魏, 学维 赵

**Affiliations:** 200003 上海，第二军医大学附属上海长征医院胸心外科 Department of Thoracic Surgery, Changzheng Hospital, the Second Military Medical University, Shanghai 200003, China

**Keywords:** 肺肿瘤, TLE1, 基因克隆, 原核表达, 蛋白纯化, 多克隆抗体, Lung neoplasms, TLE1, Gene cloning, Prokaryotic expression and purification, Polyclonal antibody

## Abstract

**背景与目的:**

TLE1是参与Wnt、Notch以及EGFR信号通路调控的一种重要蛋白。TLE1 N端Q结构域片段通过介导自身寡聚化及与LEF1结合发挥调控作用。本实验旨在构建人TLE1 N端Q区基因在大肠杆菌的融合表达载体，制备并纯化人TLE1 N端Q结构域蛋白片段，制备TLE1 Q结构域多克隆抗体。

**方法:**

以人肺腺癌cDNA库为模板聚合酶链反应（polymerase chain reaction, PCR）特异性扩增TLE1-Q(1-136)基因序列，与pGEX-4T-1质粒连接后转化感受态大肠杆菌*E.coli*。以异丙基-β-D-硫代半乳糖苷（isopropyl-β-D thiogalactoside, IPTG）诱导表达产生融合蛋白GSTTLE1-Q(1-136)。经亲和层析，Thrombin酶切，FPLC纯化，SDS-PAGE鉴定目的蛋白TLE1-Q(1-136)。免疫家兔，制备多克隆抗体。

**结果:**

测序证实重组表达质粒中的人TLE1 N端Q结构域基因序列正确，成功构建表达型重组质粒pGEX-4T1-TLE1-Q。重组质粒转化大肠杆菌C+后，经诱导，重组蛋白GST-TLE1-Q(1-136)得到表达。SDS-PAGE鉴定示纯化蛋白为目的蛋白人TLE1 N端Q结构域片段TLE1-Q(1-136)。免疫家兔后收获抗血清，ELISA显示抗体效价为1:20 000，具有高度特异性。免疫印迹结果显示，制备的多抗可与TLE1-Q(1-136)蛋白特异性结合。

**结论:**

成功构建了人TLE1 N端Q结构域重组融合蛋白表达质粒pGEX-4T1-TLE1-Q，表达纯化了稳定可溶TLE1 N端Q结构域蛋白TLE1-Q(1-136)，制备了人TLE1 N端Q结构域蛋白片段多克隆抗体，为进一步研究TLE1在肺癌生成中的作用奠定了基础。

## Abstract (en)

**Background and objective:**

TLE1 is an important protein in regulating Wnt, Notch and EGFR signaling pathways. The TLE1 N-terminal Q domain regulates the pathways by mediating its oligomerization and interaction with LEF1. The aim of this study is to construct the human TLE1 N-terminal Q domain fragment in prokaryotic expression system, express and purify protein TLE1 N-terminal Q domain and prepare its polyclonal antibody.

**Methods:**

The sequence of TLE1 N-terminal Q domain obtained by PCR from human lung adenocarcinoma cDNA, was cloned into the prokaryotic expression vector pGEX-4T-1 containing Glutathione S-transferase (GST). Vector pGEX-4T1-TLE1-Q was transformed into *E.coli* BL21 condon plus. The GST-TLE1-Q(1-136) fusion protein was induced by IPTG, digested by Thrombin, purified with glutathione-sepharose beads and FPLC, identified by SDS-PAGE. Then rabbits were immunized with the purified protein TLE1-Q(1-136) for obtaining the antiserum. The titers and specificity of antibodies were measured by ELISA and Western blot.

**Results:**

The PCR identification and the sequencing of recombinant plasmid demonstrated that vector pGEX-4T1-TLE1-Q was successfully constructed. The SDS-PAGE shows target protein (14 000 Da) is the interest protein TLE1-Q(1-136). The TLE1 N-terminal Q domain fragment TLE1-Q(1-136) and its polyclonal antibody have been acquired, with an antibody titer of 1:20 000.

**Conclusion:**

Expression vector pGEX-4T1-TLE1-Q is correctly constructed. The TLE1 N-terminal Q domain fragment TLE1-Q(1-136) and its polyclonal antibody have been acquired. These work established the foundation for further biological study between TLE1 and lung cancers.

TLE是Groucho蛋白家族在哺乳类动物中的同源物，作为转录共抑制分子，通过与Hairy、Tcf/Lef1、Runt等蛋白家族作用，形成转录抑制复合物，调控基因表达^[1-4]^。人*TLE1*基因定位于第9号染色体，全长2 310 bp，编码770个氨基酸。TLE1蛋白是Notch信号通路下游重要调控蛋白，也可参与其它多个细胞信号通路传导，如Shh、EGFR等，成为多种信号通路间相互影响、相互作用的重要靶点^[5, 6]^。TLE1蛋白通过介导细胞的分化和发育，在胚胎发育、造血细胞发育分化、神经肿瘤尤其是恶性肿瘤生成等生理病理过程中起重要作用^[7-9]^。Allen等^[8]^通过组织芯片筛选实验证实TLE1在肺腺癌中表达显著增加，又在转基因大鼠模型中发现Grg1过表达对肺腺癌生成有促进作用，因此认为TLE1与肺腺癌发生发展有密切关系。

在Groucho蛋白家族中，TLE1-4四种蛋白均在氨基端含有富含谷氨酰胺的Q结构域，在羧基端含有高度保守的特征WD40样重复的结构域，此外TLE1还有3个保守性较低的结构域，分别是GP、SP和CcN。Q结构域作为TLE1蛋白两个高度保守的特征性结构域之一，主要发挥两部分生物学作用：寡聚化作用使TLE1形成四聚体，Q结构域关键基序点突变可导致寡聚化作用失败，从而使其转录抑制功能失活^[10, 11]^；Q结构域通过直接与蛋白分子如Tcf/Lef1、PRDI结合，形成转录共轭复合物，影响下游信号蛋白表达，调控信号通路，从而发挥生物学活性^[12, 13]^。本实验通过对人TLE1 N端Q结构域的表达纯化，得到高纯度的Q结构域蛋白片段TLE1-Q(1-136)，并成功制备其多克隆抗体，为解析TLE1-Q结构域蛋白结构、明确TLE1-Q结构域在Notch信号通路的作用方式、了解TLE1调控多条信号通路机理、理解TLE1促进肺癌生成机制打下基础。

## 1 材料和方法

### 1.1 菌株和表达载体

人肺腺癌cDNA文库（由本室前期保存）；大肠杆菌*E.coli* BL21 condon plus及pGEX-4T-1载体由中科院上海生命科学院结构生物学平台杜嘉木博士提供。

### 1.2 主要试剂

DNA胶回收试剂盒和质粒抽提试剂盒购自天根生化科技（北京）有限公司；KOD plus DNA聚合酶、dNTP、限制性内切酶*Bam*HI和*Xho*lⅠ连接酶购自宝生物工程（大连）有限公司；Ligation High DNA连接酶购自东洋纺（上海）生物科技有限公司；Glutathion Sepharose 4B Fast Flow亲和层析凝胶、快速蛋白液相色谱（FPLC）、Thrombin酶购自GE公司；纯种新西兰大白兔6只（雄性）购自第二军医大学实验动物中心。

### 1.3 引物设计

根据GenBank中人类TLE1基因序列（NM_005077），Omiga 2.0设计引物，选取TLE1 N端第1-136氨基酸残基，设计以*Bam*HI、*Xho*lⅠ为酶切位点。引物序列为：tle5’: ata gga tcc atg ttc ccg cag agc和tle3’: tat ctc gag tca gcc atg aga aag atg。

### 1.4 基因扩增

以人肺腺癌cDNA库为模板，以tle5’和tle3’作引物，KOD plus DNA聚合酶扩增。PCR反应参数设置为94 ℃预变性5 min，94 ℃变性30 s，55 ℃退火30 s，68 ℃延伸30 s，30个循环后，68 ℃延伸10 min，4 ℃孵育20 min。

### 1.5 重组表达载体的构建及鉴定

人类TLE1 N端Q区基因PCR扩增产物以1%琼脂糖凝胶电泳检测，以QIANGEN胶回收试剂盒，按标准说明书回收。回收产物及质粒载体pGEX-4T-1以*Bam*HI和*Xho*lⅠ限制性内切酶37 ℃酶切过夜。回收的酶切产物TLE1 N末端基因与pGEX-4T-1质粒以Ligtion High DNA连接酶16 ℃连接过夜。重组表达载体转化感受态细胞*E.coli* BL21 condon plus，Amp抗性筛选重组阳性菌，命名为pGEX-4T1-TLE1-Q，PCR及*Bam*HI、*Xho*lⅠ双酶切鉴定，送上海博尚生物公司测序。

### 1.6 TLE1 N端Q结构域的表达纯化

pGEX-4T1-TLE1-Q菌株于LB 37 ℃培养至OD≈0.8，以0.5 mmol/L IPTG，16 ℃诱导过夜。离心收集菌体，以PBS（pH8.0）为破菌缓冲液，超声波破菌。高速离心收集上清液，与Glutathion Sepharose 4B Fast Flow beads混合3 h，PBS（pH8.0）洗脱未结合蛋白，10 mmol/L还原性谷胱甘肽洗脱融合目的蛋白GST-TLE1-Q(1-136)。100 U Thrombin 4 ℃，酶切16 h，透析至PBS（pH8.0），再次亲和纯化，去除GST-tag。以PBS 0.1%Chaps（pH8.0）洗脱目的蛋白TLE1-Q(1-136)，FPLC纯化，SDS-PAGE分析纯度。

### 1.7 多克隆抗体的制备

以纯化蛋白分别免疫新西兰大白兔，将300 μg TLE1-Q(1-136)蛋白与完全弗氏佐剂等体积混合充分乳化后，背部皮下多点注射初次免疫，21 d后200 μg TLE1-Q(1-136)蛋白与等体积的不完全弗氏佐剂混合乳化后，颈背部皮下多点加强免疫，加强免疫2次后，采心脏血，制备抗血清分离血清，分装储存于-20 ℃保存。间接ELISA法检测多克隆抗体效价。

### 1.8 Western blot分析

以pGEX-4T-1空载体转化的*E.coli* BL21 condon plus为阴性对照，纯化的TLE1-Q(1-136)蛋白经SDS-PAGE电泳，电转移（100 V，2 h，冰水浴）至硝酸纤维膜上，5%脱脂奶粉封闭，将制备的多克隆抗体（1:1 000稀释），4 ℃孵育过夜。1‰TBST洗膜，10 min/次，共3次，加入带HRP标记的羊抗兔二抗（1:2 000稀释），室温下孵育1 h，1‰TBST洗膜，15 min/次，共3次，最后加入化学荧光试剂，压片，曝光，拍照。

## 2 结果

### 2.1 TLE1 N端Q区基因扩增鉴定

以构建的pGEX-4T1-TLE1-Q质粒载体为模板，以tle3’及tle5’为引物PCR扩增，双酶切后分别出现大小约408 bp的特异性条带，与目的大小片段相一致（图 1）。测序结果显示所获得的TLE1第1-136位氨基酸残基样品序列编码框架完整，编码区为408 bp，编码136个氨基酸残基。经Pubmed网站Blast分析，与人类基因组*TLE1*基因序列同源性为100%，编码TLE1蛋白N末端第1-136个氨基酸。

**1:**
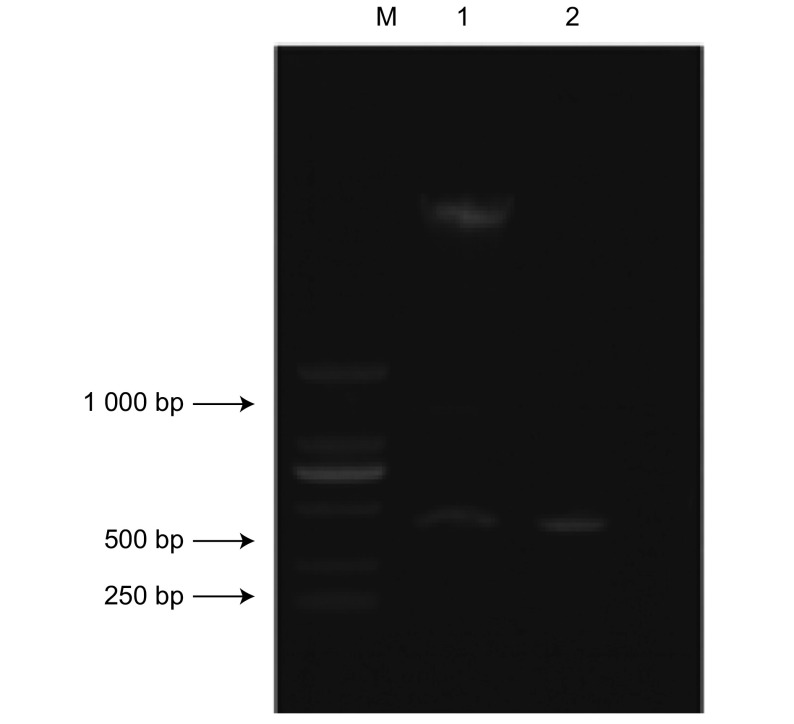
重组质粒pGEX-4T1-TLE1-Q的PCR及双酶切鉴定 Identification of vector pGEX-4T1-TLE1-Q by PCR and digested with *Bam*HI and *Xho*lⅠ

### 2.2 重组人TLE1 N端Q结构域蛋白的表达及纯化结果

目的TLE1-Q(1-136)蛋白表达纯化后，以13.5%SDS-PAGE对全菌，上清，洗脱、酶切及纯化产物蛋白行鉴定分析。SDS-PAGE表明经一步亲和纯化，还原型谷胱甘肽洗脱，可以得到分子量约为41 000 Da左右的重组蛋白GST-TLE1-Q(1-136)。Thrombin酶切后，41 000 Da目的条带切开成为大小约26 000 Da和14 000 Da两条条带。再次经亲和、FPLC纯化，可得到大小约14 000 Da的目的蛋白TLE1-Q(1-136)（图 2）。

**2:**
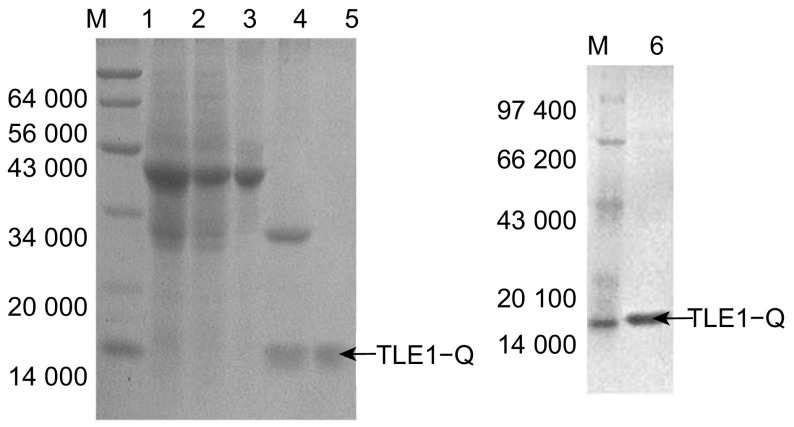
重组蛋白GST-TLE1-Q(1-136)的SDS-PAGE鉴定 SDS-PAGE of recombinant protein GST-TLE1-Q(1-136)

### 2.3 TLE1 N端Q结构域蛋白多克隆抗体效价及特异性测定

纯化的TLE1 N端Q结构域蛋白TLE1-Q(1-136)以（0.1 μg/mL）每孔100 μL包被，ELISA法检测多克隆抗体效价，制备的抗血清效价达1:20 000。对TLE1-Q(1-136)蛋白经自制抗体行免疫印迹检测，可见一条分子量约14 000 Da的清晰条带，与预期结果相符，阴性对照未见明显条带，证明制备的抗体特异性较好（图 3）。

**3:**
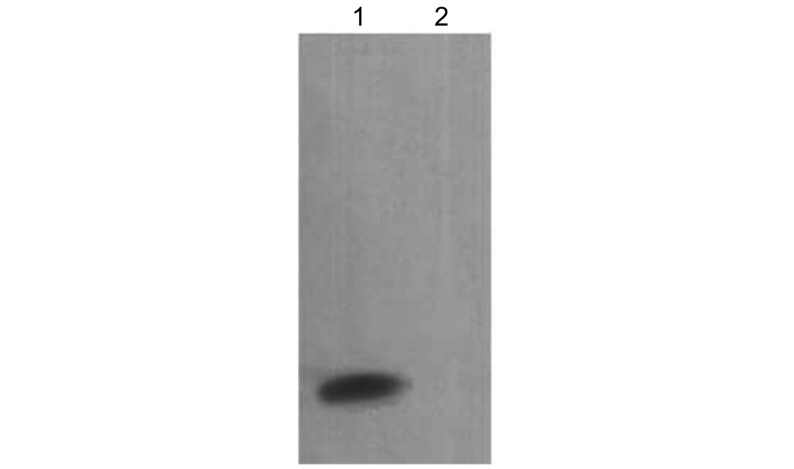
TLE1-Q(1-136)多克隆抗体的Western blot鉴定 Identification of polyclonal antibody by Western blot

## 3 讨论

Gro/TLE家族作为转录共抑制分子，本身不与DNA直接结合，通过结合转录抑制蛋白形成复合物，对靶基因起“长程”转录抑制作用^[4, 14]^。Groucho/TLE蛋白分子中具有两个高度保守的特征性结构域，分别是：位于羧基端的含有多个色氨酸-天门冬氨酸二肽序列的WD40结构；位于氨基端的由130个氨基酸组成、富含谷氨酰胺的Q结构域。前期研究认为TLE1通过WD结构域与WRPW、Eh基序结合形成复合物，该复合物经N-末端由第1-136位氨基酸组成的Q结构域来发挥其转录抑制活性，可能机制为TLE1通过位于Q结构域的2个亮氨酸拉链状的α螺旋，形成四聚体抑制转录活性^[10]^。实验证实将亮氨酸突变后，TLE/Gro无法形成四聚体，失去转录抑制活性，分析认为TLE分子中第50-110位氨基酸残基对四聚体的形成起到关键作用^[11]^。近来亦有研究^[13, 15]^表明Q结构域不仅介导TLE及其家族蛋白之间的四聚化作用，也可直接结合Tcf/Lef1、gp130等转录调控因子，参与信号通路调控。本实验即选取TLE1第1-136位氨基酸残基TLE1-Q(1-136)即Q结构域所在片段进行表达纯化研究。

在原核系统中表达真核蛋白，蛋白的正确折叠是难点之一。蛋白的可溶性是由蛋白结构的空间折叠方式决定的，不同的折叠方式也可影响到蛋白分子上抗原决定簇，进而影响抗体的特异性与效价^[16]^。由于富含谷氨酰胺的蛋白在溶液中的可溶性较差^[17]^，我们通过16 ℃低温诱导，控制蛋白生成速度，使目的蛋白以合适的速度转录、翻译，从而使其正确折叠，并借助GST-tag的助溶性，在上清中形成可溶的重组蛋白；同时GST-tag也为蛋白的纯化提供了亲和位点。SDS-PAGE电泳证实获得较高纯度的可溶重组蛋白GST-TLE1-Q(1-136)，相对分子质量约为41 000 Da。由于无GST-Tag的TLE1蛋白的Q结构域性状更接近体内真实天然状态，并利于后期研究；且GST标签（26 000 Da）较Q结构域（14 000 Da）更大，用重组蛋白在制备抗体过程中可能会造成抗体的特异性和效价降低。我们尝试以Chaps为去垢剂，帮助维持蛋白在溶液中的稳定溶解状态，并以Thrombin酶切去除GST标签，最终得到相对分子质量约为14 000 Da、纯度高、特异性强、稳定可溶的TLE1N端Q结构域蛋白TLE1-Q(1-136)。用该纯化蛋白免疫家兔，成功制备该蛋白的多克隆抗体，ELISA检测效价为1:20 000，经Western blot与纯化蛋白TLE1-Q(1-136)免疫杂交出现条带，与预期结果一致，证实我们抗体制备成功，且具有高度的特异性。

TLE/Gro参与Shh、EGFR、Notch等多个信号通路的活化后调控，在信号通路活化、高等动物胚胎发育等生理病理过程中起到重要作用^[4, 6]^。TLE1作为以上信号通路的关键调控点，重要机制之一就是通过TLE1 N端Q结构域结合Tcf/Lef1、FoxA、c-Myc、PRDI等相关蛋白而引起转录调节因子活性的改变，或介导四聚体形成转录抑制复合物，实现对目的基因转录的调控，引起胚胎发育，肿瘤增殖等一系列病理生理变化，但其具体分子作用机制尚不十分明晰。本实验研究成功制备可溶TLE1 N端Q结构域蛋白TLE1-Q(1-136)及其多克隆抗体，为下一步开展针对TLE1 Q结构域的蛋白结构功能研究，了解TLE1参与信号通路调控机制，探讨TLE1在肺癌的发生发展中的作用奠定了前期研究基础。
